# Root and Root Canal Anatomy of Primary Mandibular Central Incisor, Lateral Incisor, and Canine in Indian Children: A Cone Beam Computed Tomography Study

**DOI:** 10.1155/2022/7191134

**Published:** 2022-03-07

**Authors:** Farhin Katge, Uma B. Dixit

**Affiliations:** Pedodontics and Preventive Dentistry, D. Y. Patil University-School of Dentistry, Navi Mumbai 400 706, India

## Abstract

**Background:**

A thorough knowledge of root and root canal morphology in primary dentition is essential for success of endodontic therapy. This information also finds importance in anthropological research in reconstructing human population history. Lack of studies of root and root canal morphology in mandibular anterior teeth prompted us to the present study.

**Methods:**

A total of 109 extracted primary mandibular incisors and canines were collected, out of which 90 teeth were selected for this study and divided into 3 groups: CI, mandibular central incisor; LI, mandibular lateral incisor; C, mandibular canine. All the sample teeth were scanned using cone beam computed tomography (CBCT). Number of roots, number of root canals, length of root, mesiodistal (MD), and buccolingual (BL) width of canal, shape of canal, and presence of accessory canals were assessed. Collected data were statistically compared using one-way ANOVA and chi-square tests.

**Results:**

All teeth studied displayed single root with single root canal conforming to type I Vertucci's classification. Root length of CI was significantly shorter than both LI and C, with no significant difference between LI and C. Straight root canals were more common in CI and LI, whereas curved canals were more common in C. S-shaped canals were seen in a few CI and C. BL canal width was more than MD width in all teeth, C showing significantly larger dimensions than both CI and LI.

**Conclusion:**

This study presents root and root canal characteristics of primary mandibular central incisor, lateral incisor, and canine in children from Indian ethnicity.

## 1. Introduction

Preservation of primary teeth with pulpal disease not only maintains space but also restores the teeth to normal function. Retention of primary teeth allows normal shedding and continued bone development and subsequent eruption of the permanent successor.

Primary mandibular canine is often affected by caries during early childhood and consequently may show pulpal damage. Premature extraction of these canines may lead to midline shift when performed unilaterally [1]. Bilateral extractions of primary mandibular canines may lead to retrusion of the lower incisors and loss of arch length, especially in crowded arches [2, 3]. To avoid these complications and further orthodontic treatment, the clinician may decide against extraction.

Pulpectomy remains the choice of treatment in pulpally affected primary teeth where roots and surrounding bone show minimal damage. The objective of pulpectomy procedure of primary teeth is to maintain integrity and health of teeth and supporting tissues [4]. Pulpectomy procedure includes debridement of pulpal tissue and cleaning and shaping of the canals, followed by obturation with a suitable material.

The root canal system in primary dentition is shown to have a wide range of unpredictable anatomical variations [5–7]. As primary teeth may show unusual internal geometry of the pulpal cavity with features not commonly observed in permanent teeth, such as connections to furcation and horizontal anastomoses, endodontic treatment of primary teeth is considered highly complicated [8]. A thorough knowledge of the root canal system of primary teeth is essential to perform successful endodontic treatment. Poor knowledge of root canal morphology and inability to identify root canal orifice or track down the ribbon shape canals are some of the factors which may lead to failure of endodontic procedure in pediatric patient.

Accurate knowledge of the roots and root canal system of primary teeth can provide a vital source of information for anthropological research, as it is found that both show considerable variation according to geography and ethnicity [9].

Many researchers such as Ahmed et al., Ozcan et al., Reddy et al., Neboda et al., and El Hachem et al. have contributed to knowledge and understanding of the root canal system in primary teeth [7, 10–13].

Root canal morphology of primary teeth has been studied by using the dye injection technique, dye perfusion technique, digital radiographs, cross-sectioning technique, histological examinations, clearing technique, and many more [6, 13, 14]. These techniques have been successfully used for many years in the morphological study of the root canal system in both primary and permanent dentition. However, most of them are invasive, technique sensitive, and only provide a two-dimensional (2D) image of a three-dimensional (3D) structure and therefore might not directly reflect the actual morphology of teeth being studied. Recently, to overcome these shortcomings, cone beam computed tomography (CBCT) has been used to study root canal morphology.

Zoremchhingi et al., Ozcan et al., Yang et al., and Datta et al. studied root canal morphology of primary maxillary and mandibular molars using CBCT [10, 15–17]. However, there are limited studies in literature assessing root canal morphology of primary mandibular anterior teeth [18, 19]. Hence, the aim of this study was to study root and root canal morphology of primary mandibular central incisor, lateral incisor, and canine using CBCT.

## 2. Materials and Methods

This cross-sectional study was approved by the institutional research and ethical board (IREB/2021/PHD/PEDO/01).

### 2.1. Sample

Extracted human primary mandibular incisors and canines were used for this study. Primary mandibular incisors and canines that were extracted by dentists from more than 20 dental colleges and hospitals from various regions in India were obtained. The reasons for extraction were unknown to authors and were not related to the present study.

Sample size was calculated based on root length data of primary mandibular incisors from an earlier study by Gaurav et al. (mean ± SD: 9.52 ± 1.34) [18]. Using 5% error and 95% confidence interval, sample size was estimated to be 30. As we aimed to study characteristics of central incisors, lateral incisors, and canines separately, we estimated total sample size of 90 teeth, 30 each of primary mandibular central incisors, lateral incisors, and canines.

### 2.2. Selection Criteria

Extracted primary mandibular incisors and canines with no evidence of root resorption were selected for this study. Teeth with root fracture, internal root resorption, external root resorption, canal obliteration, restored teeth, and dental anomaly of shape, size, and structure were excluded.

From the total of 109 collected primary mandibular incisors and canines (36 central incisors, 35 lateral incisors, and 38 canines), 90 teeth (30 each of central incisors, lateral incisors, and canines) were selected randomly and accordingly assigned to three groups: CI (mandibular central incisor), LI (mandibular lateral incisor), and C (mandibular canine).

## 3. Procedure

**Figure 1:**
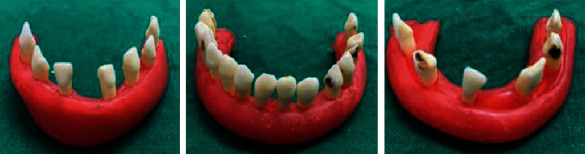
Sample teeth mounted on modelling wax in arch form.

Included teeth were cleaned ultrasonically and stored in a glass container containing saline solution at room temperature. The sample teeth were then mounted on modelling wax in an arch form after determining various aspects of the tooth: buccal, lingual, mesial, and distal, so as to maintain uniformity in the samples (Figure 1).

The teeth were then scanned using the CBCT machine (NewTom, Giano/VG3, Imola, Italy) operating at 90 KVp and 10.80 mA with field of view (FoV) 11 × 5 cm voxel size of 300 microns.

Volume rendering and 3D images were reconstructed using the NNT viewer software (NewTom, version 10.0 Imola, Italy). Two independent expert pediatric dentists with clinical and academic experience of more than 20 years assessed all CBCT images in three planes: sagittal, axial, and coronal. Both the examiners were trained using the CBCT software. CBCT images were viewed and analyzed using Dell Inspiron 3891 desktop and Dell E-series 24-inch HD screen with 1920×1080 resolution (Dell, Round Rock, USA). Following observations were recorded for each tooth.

**Figure 2:**
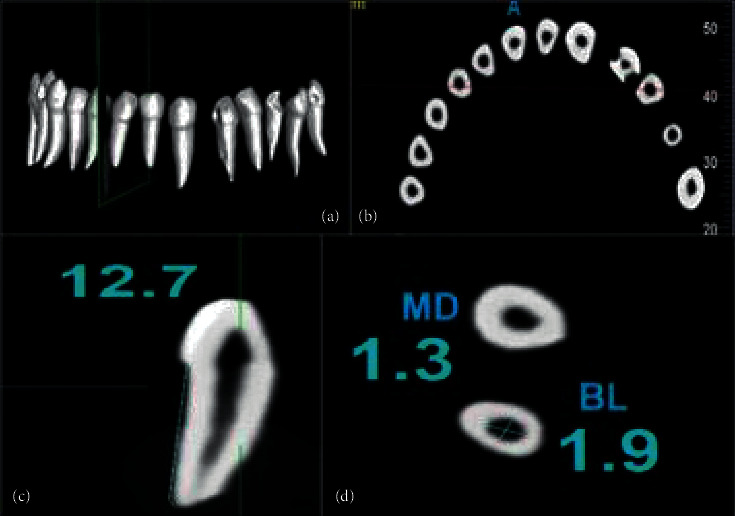
(a) Number of roots. (b) Number of root canals. (c) Length of root. (d) Width of root canal.

**Figure 3:**
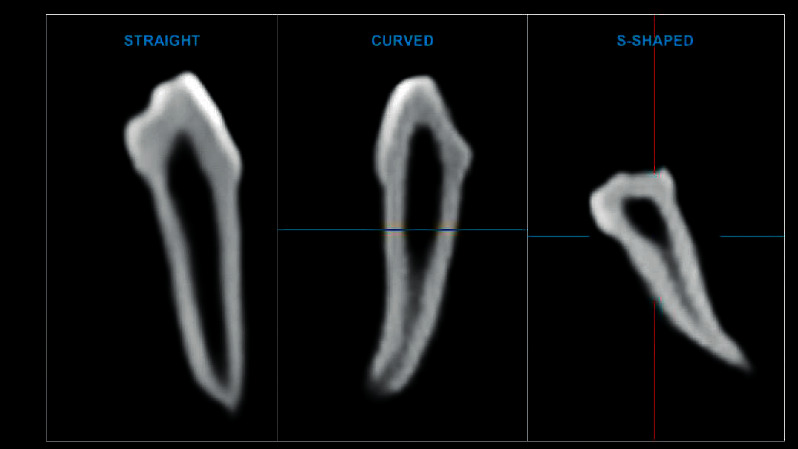
Shape of root canal.

1. Number of roots per tooth was counted manually on 3D reconstructed images (Figure 2(a))
2. Number of root canals in each root was counted after assessing coronal and sagittal section of CBCT images (Figure 2(b))
3. Morphology of the root canal system was classified according to Vertucci's classification [20]. Root canal morphologies not listed in Vertucci's classification were noted down according to their forms.
4. Presence, location, and number of accessory canals and intercanal communications were recorded using axial, coronal, or sagittal section of CBCT images.
5. Length of each root was measured using the measurement tool in the NNT viewer software by taking the maximum length from apex of the tooth to the greatest area of constriction at cementoenamel junction (CEJ) on coronal or sagittal section of CBCT image (Figure 2(c)).
6. Width of root canal of each root was measured at 2 mm from CEJ on axial section of CBCT image. The line was drawn from mesial aspect to distal aspect of inner root canal wall, and this was calculated as mesiodistal (MD) width of the root canal. Similarly, buccolingual (BL) width of the canal was calculated from buccal aspect to lingual aspect of inner root canal wall (Figure 2(d)).
7. Shape of each root canal was categorized as straight, curved, or S-shaped on sagittal section of CBCT image (Figure 3)

### 3.1. Statistical Analysis

The data obtained were tabulated and analyzed statistically using SPSS version 17.0 (SPSS, Inc., Chicago, IL). Descriptive statistics were performed to calculate the frequency of categorical variables and mean, standard deviation, and range for continuous variables. Intergroup comparison of continuous variables was performed using the one-way ANOVA test. Whenever significant, Bonferroni analysis was used for post-hoc comparisons. The chi-square or Fisher's exact test was used to evaluate association between the type of tooth and categorical variables. Significance was set at 0.05. Interexaminer and intraexaminer reliabilities were calculated using interclass correlation coefficient (ICC). The ICC value less than 0.5 is indicative of poor reliability, value between 0.5 and 0.75 indicates moderate reliability, value between 0.75 and 0.9 indicates good reliability, whereas value greater than 0.9 indicates excellent reliability.

## 4. Results

A total of 90 extracted primary mandibular single-rooted teeth (30 each of central incisors, lateral incisors, and canines) were scanned using CBCT and analyzed for this study. The ICC for interexaminer reliability and intraexaminer reliability was 1 for number of roots, number of root canals, and root canal morphology. In all the three groups, ICC values for interexaminer reliability were good for length of root, whereas the values for intraexaminer reliability were excellent. ICC values of both interexaminer and intraexaminer reliabilities were good in all three groups for BL width and MD width of root canal (Table 1).

All teeth displayed single roots with one root canal per root. Root canal morphology of all the teeth conformed to type I Vertucci's classification: presence of a single main canal starting from the pulp chamber to root apex. None of the teeth showed presence of accessory canals.

Root length of C was found to be significantly longer than CI (*p* < 0.001, Table 2) and statistically similar to LI (*p*=0.56). Root length of LI was significantly more than CI (*p*=0.03).

In all the teeth studied, BL canal width was more than MD canal width (Table 2). In CI, mean MD width of canal, measured at 2 mm below CEJ, was found to be 1.35 ± 0.39 mm (range: 0.9–2.2) and mean BL width was 1.43 ± 0.37 mm (range: 0.6–2.1). In LI, the mean value of MD canal width was 1.4 ± 0.31 mm (range: 0.9–1.8) and BL canal width was 1.59 ± 0.32 mm (range: 1.2–2). In C, mean MD canal width was found to be 1.82 ± 0.31 mm (range: 1.3–2.4) and mean BL width was 1.92 ± 0.28 mm (range: 1.5–2.3).

Intergroup comparison revealed that MD canal width of C was significantly more than that of CI and LI (both *p* < 0.001). No statistical difference was found in MD canal width between CI and LI (*p*=1.0). Similarly, BL canal width of C was significantly more than that of CI and LI (both, *p* < 0.001), with no significant difference found between CI and LI (*p*=0.18).

**Figure 4:**
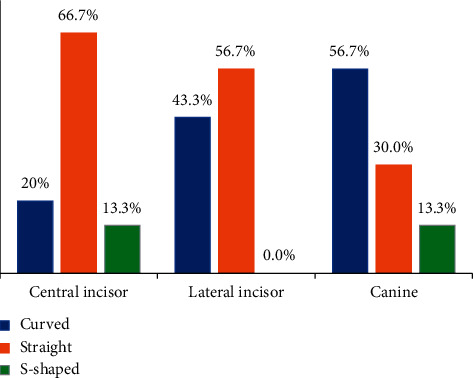
Comparison of shape of root canals between the three groups.

Straight canal was found in 20 CI (66.7%), 17 (56.7%) LI, and only 9 C (30%) (Figure 4). More C (17, 56.7%) showed curved canals, followed by LI (13, 43.3%) and CI (6, 20%). S-shaped canals were seen in 4 CI (13.3%) and 4 C (13.3%), whereas none of the LI had S-shaped canals. A significant association was found between the type of tooth and shape of canal (*χ*^2^ = 13.38, *p*=0.01, Cramer's V = 0.27).

## 5. Discussion

The present study provides a detailed descriptive analysis about root and root canal morphology of primary mandibular central incisors, lateral incisors, and canines in Indian children. CBCT images of extracted teeth allowed 3D visualization of external and internal morphologies of teeth.

CBCT is a nondestructive method, which allows 3D reconstruction and visualization of external and internal morphologies of teeth. It is considered to be highly accurate [21]. Hence, CBCT was selected as an assessment tool for the present study.

To the best of our knowledge, there is only one study that studied root canal morphology of primary mandibular incisors in Indian children using CBCT (Gaurav et al.) [18]. One of the shortcomings of their study was that the authors did not specify the type trait (central or lateral) of mandibular incisors. Surprisingly, our literature search did not reveal any studies on root canal morphology of primary mandibular canine, although pulpectomy of these teeth is a routinely followed procedure in pediatric dental practices [22, 23].

We found that root length of CI was significantly shorter than LI and C. When compared to the table of measurements given in the “Wheeler's Dental Anatomy, Physiology and Occlusion” by Nelson and Ash, root length of C in our study (11.8 mm) was similar (11.5 mm); whereas, root lengths of CI and LI in our sample were longer (CI: 10.25 vs. 9.0 mm, LI: 11.28 vs. 10.0 mm) [24]. In 2012, Charles Goodacre, in his Atlas of the human dentition, presented root length measurements of primary teeth calculated from six sources from literature (Black, Dewey, Kramer and Ireland, Linek, Ash, and Woelfel and Scheid) [25–31]. The mean values of root lengths estimated by him were CI: 9.2 mm, LI: 10.0 mm, and C: 11.3 mm. The C root length mean was similar to that estimated by Goodacre; however, CI and LI root lengths were longer. In the study by Gaurav et al. for Indian population, they reported shorter root lengths (9.5 mm) in primary mandibular incisors (combined central and lateral incisors) as compared to our study [18]. The variation in root length characteristics may be explained by variation in factors such as geography, ethnicity, sex, and genetics [32].

Our findings revealed that all the studied teeth, despite their type trait, had single root with single root canal. Similar observation has been reported earlier in literature [24, 32, 33]. However, Zurcher reported the presence of two canals in less than 10% of primary mandibular incisors [34]. Primary mandibular canines with bifurcated roots and bifurcated root canals have been reported in occasional case reports [35–37]. Such variations were not observed in our sample.

Our finding that root canal morphology of all 90 teeth belonged to type I Vertucci's classification, despite the type trait is in contradiction to findings given by Gaurav et al. who reported type I canal morphology (single canal) in 87% incisors studied and type III (single canal with bifurcation in the middle third) in 13% of teeth [18]. Our sample size for primary mandibular incisors was much larger (60) than used in their study (15). All available descriptions of root canal morphology of primary mandibular incisors in the literature refer to single root canal [32].

Diameter of the root canal has been studied earlier by passing a line through centre of the canal and parallel to mesiodistal and buccolingual plane in previous studies [5, 13, 15, 18]. However, root canals are not circular in shape; in most instances, they may be oval, ribbon-shaped, or C-shaped [5, 38, 39]. Therefore, in the present study, rather than a diameter, MD and BL width of the canal were considered. We found that most canals were oval-shaped, with BL width more than the MD width. Fumes et al. observed oval-shaped canals in primary maxillary molars [5].

Straight root canals were more commonly seen in CI and LI. Curved canals were observed more frequently in C, whereas S-shaped canal was observed in CI and C.

This present study has added important information to the current knowledge of morphology of roots and root canals of primary mandibular incisors and canines. Understanding root canal morphology is of utmost importance to prevent problems such as ledge formation, endodontic file separation, perforation, or canal transportation during the pulpectomy procedure. Also, root and root canal morphologies of population from specific geographical area or ethnicity would help in reconstructing human population history, especially related to direction of migration and genetic relationship.

## 6. Conclusion

Cone beam computed tomography can be considered as a simple, highly accurate, and reliable tool for studying root and root canal morphology. All primary mandibular central incisors, lateral incisors, and canines included in this study had single roots with single canal per root. Canine root was significantly longer than central incisor, central incisor root being significantly shorter than both lateral incisor and canine. Root canal morphology of all teeth was classified as type I Vertucci's classification. Buccolingual width of the canal was more than mesiodistal width in all the teeth. Both buccolingual and mesiodistal canal widths of canine were significantly larger than that of central incisors and lateral incisors. Straight root canals were predominantly seen in central and lateral incisors, whereas curved root canals were commonly seen in canines. S-shaped canals were seen in a few central incisors and canines, whereas none of the lateral incisors exhibited S-shaped canals. Significant association was found between the type of tooth and shape of canal. None of the teeth showed the presence of accessory canals.

Findings from the present study will help clinicians to enhance their knowledge regarding root and root canal morphology of primary mandibular central incisor, lateral incisor, and canine.

## Figures and Tables

**Table 1:** Interexaminer and intraexaminer reliabilities of length of root, MD, and BL widths of root canals.

Parameters	Groups	Interclass correlation coefficient	
		Interexaminer reliability	Intraexaminer reliability
Length of root (mm)	CI	0.87	0.91
	LI	0.89	0.91
	C	0.89	0.92

MD width of the root canal (mm)	CI	0.77	0.81
	LI	0.80	0.83
	C	0.79	0.86

BL width of the root canal (mm)	CI	0.78	0.84
	LI	0.81	0.83
	C	0.86	0.89

**Table 2:** Intergroup comparison of length of root, MD, and BL widths of root canals.

Characteristics	Mean ± SD (min, max)			Statistical comparison
	CI	LI	C	
Length of root (mm)	10.25 ± 1.33 (8.2, 11.9)	11.28 ± 1.81 (8.4, 13.1)	11.8 ± 1.41 (8.8, 14.2)	ANOVA *F* = 8.02, *p*=0.001 CI vs. LI: 0.03 CI vs. C: <0.001 LI vs. C: 0.56
MD width of the root canal (mm)	1.35 ± 0.39 (0.9, 2.2)	1.4 ± 0.31 (0.9, 1.8)	1.82 ± 0.31 (1.3, 2.4)	ANOVA *F* = 17.6, *p* < 0.001 CI vs. LI: 1.0 CI vs. C: <0.001 LI vs. C: <0.001
BL width of the root canal (mm)	1.43 ± 0.37 (0.6, 2.1)	1.59 ± 0.32 (1.2, 2.0)	1.92 ± 0.28 (1.5, 2.3)	ANOVA *F* = 17.89, *p* < 0.001 CI vs. LI: 0.18 CI vs. C: <0.001 LI vs. C: <0.001

## Data Availability

Data can be made accessible to the interested researchers by the corresponding author on reasonable request.
